# HIV-1 Vpr stimulates NF-κB and AP-1 signaling by activating TAK1

**DOI:** 10.1186/1742-4690-11-45

**Published:** 2014-06-09

**Authors:** Ruikang Liu, Yongquan Lin, Rui Jia, Yunqi Geng, Chen Liang, Juan Tan, Wentao Qiao

**Affiliations:** 1 Key Laboratory of Molecular Microbiology and Biotechnology (Ministry of Education) and Key Laboratory of Microbial Functional Genomics (Tianjin), College of Life Sciences, Nankai University, Tianjin 300071, China; 2 Lady Davis Institute, Jewish General Hospital, Montreal, Qc H3T 1E2, Canada; 3 Departments of Medicine and Microbiology and Immunology, McGill University, Montreal, Qc, Canada; 4 Microbiology and Immunology, McGill University, Montreal, Qc, Canada

**Keywords:** Vpr, TAK1, Phosphorylation, Polyubiquitination

## Abstract

**Background:**

The Vpr protein of human immunodeficiency virus type 1 (HIV-1) plays an important role in viral replication. It has been reported that Vpr stimulates the nuclear factor-κB (NF-κB) and activator protein 1 (AP-1) signaling pathways, and thereby regulates viral and host cell gene expression. However, the molecular mechanism behind this function of Vpr is not fully understood.

**Results:**

Here, we have identified transforming growth factor-β-activated kinase 1 (TAK1) as the important upstream signaling molecule that Vpr associates with in order to activate NF-κB and AP-1 signaling. HIV-1 virion-associated Vpr is able to stimulate phosphorylation of TAK1. This activity of Vpr depends on its association with TAK1, since the S79A Vpr mutant lost interaction with TAK1 and was unable to activate TAK1. This association allows Vpr to promote the interaction of TAB3 with TAK1 and increase the polyubiquitination of TAK1, which renders TAK1 phosphorylation. In further support of the key role of TAK1 in this function of Vpr, knockdown of endogenous TAK1 significantly attenuated the ability of Vpr to activate NF-κB and AP-1 as well as the ability to stimulate HIV-1 LTR promoter.

**Conclusions:**

HIV-1 Vpr enhances the phosphorylation and polyubiquitination of TAK1, and as a result, activates NF-κB and AP-1 signaling pathways and stimulates HIV-1 LTR promoter.

## Background

Human immunodeficiency virus type 1 (HIV-1) codes an accessory protein called viral protein R (Vpr) that all primate lentiviruses have [1,2]. Vpr is a 14-kDa protein, and is found in both the cytoplasm and the nucleus of infected cells [3]. Notably, Vpr is specifically incorporated into progeny virions through interacting with viral Gag protein [4-6]. This presence in HIV-1 particles enables Vpr to play important roles at the early stage of viral infection, such as promoting the nuclear transport of the viral pre-integration complex (PIC) and enhancing the fidelity of the reverse transcription [7,8]. In addition, Vpr has also been reported to induce cell cycle G_2_/M arrest, regulate apoptosis, transactivate HIV-1 LTR and affect the production of IL-6, IL-8, and CCL5 [9-18]. These diverse functions of Vpr partially result from its ability to modulate the activities of cellular factors such as nuclear factor-κB (NF-κB) and activator protein 1 (AP-1) that are not only essential for multiple important cellular processes but also for HIV-1 gene expression [16-19]. However, it is not fully understood how Vpr exerts its effect on NF-κB and AP-1.

The activities of NF-κB and AP-1 are regulated by a couple of signaling pathways in response to various stimuli such as cytokines, bacterial and viral infections. These signal transduction cascades share a key regulator called TAK1 (transforming growth factor-β-activated kinase 1), a member of MAPK kinase kinase (MAP3K) family, that responds to different stimuli and assembles into a complex including the receptor proximal complex containing TNF receptor associated factor (TRAF), TAK1 binding protein 1, 2 and 3 (TAB1, TAB2, and TAB3) [20-26]. Among these TAK1-binding proteins, TAB1 interacts constitutively with the NH2-terminal catalytic domain of TAK1 and regulates the oligomerization and auto-phosphorylation of TAK1 [27-31]. TAB2 or TAB3, binds to the COOH-terminal region of TAK1, and functions as an adaptor protein to recruit TAK1 to the K63-linked polyubiquitin chains formed by TRAF6 [24,26,32-35]. TAK1 then initiates the MKK cascade by activating c-Jun N-terminal kinase (JNK) and p38 MAPK, and stimulates the IκB kinase (IKK) pathway that leads to NF-κB activation [25,36].

The activity of TAK1 is regulated by phosphorylation and polyubiquitination. Upon cytokine stimulation, TAK1 undergoes auto-phosphorylation as a result of association with TAB2/3-polyubiquitin chains [37] or TAB1-dependent oligomerization [30,38]. Phosphorylation occurs at four conserved serine and threonine residues within TAK1 activation loop, including Thr-178, Thr-184, Thr-187, and Ser-192 [28,30,31,39,40], among which Thr-187 phosphorylation has a major role in regulating TAK1 activity [30,31]. TNFα, IL-1β, and TGFβ cause K63-linked polyubiquitination of TAK1 by TRAF2 or TRAF6 [41,42]. The ubiquitin acceptor sites have been mapped to Lys-34, Lys-158, or Lys-209 in a stimulus-specific manner [41,43-47]. The possible interplay between phosphorylation and polyubiquitination of TAK1 is unclear [37].

In light of its key roles in different signaling pathways, it is not surprising that TAK1 has become the target of many viruses in order to modulate the production of pro-inflammatory cytokines expression for the benefit of virus replication. In the case of HIV-1, the viral gp41 protein has been reported to induce TAK1-dependent NF-κB signaling which enhances viral replication in CD4+ T cells [48]. In this study, we further show that the HIV-1 virion-associated Vpr activates NF-κB and AP-1 via TAK1-dependent pathways. This activity of Vpr results from its association with the TAK1 and consequent stimulation of TAK1 phosphorylation and polyubiquitination. Our findings not only highlight the essential role of TAK1 in regulating the NF-κB and AP-1 signaling cascades, but also present one example in regard to how pathogens assimilate TAK1 to enhance their multiplication.

## Results

### Vpr induces the phosphorylation of TAK1

**Figure 1:**
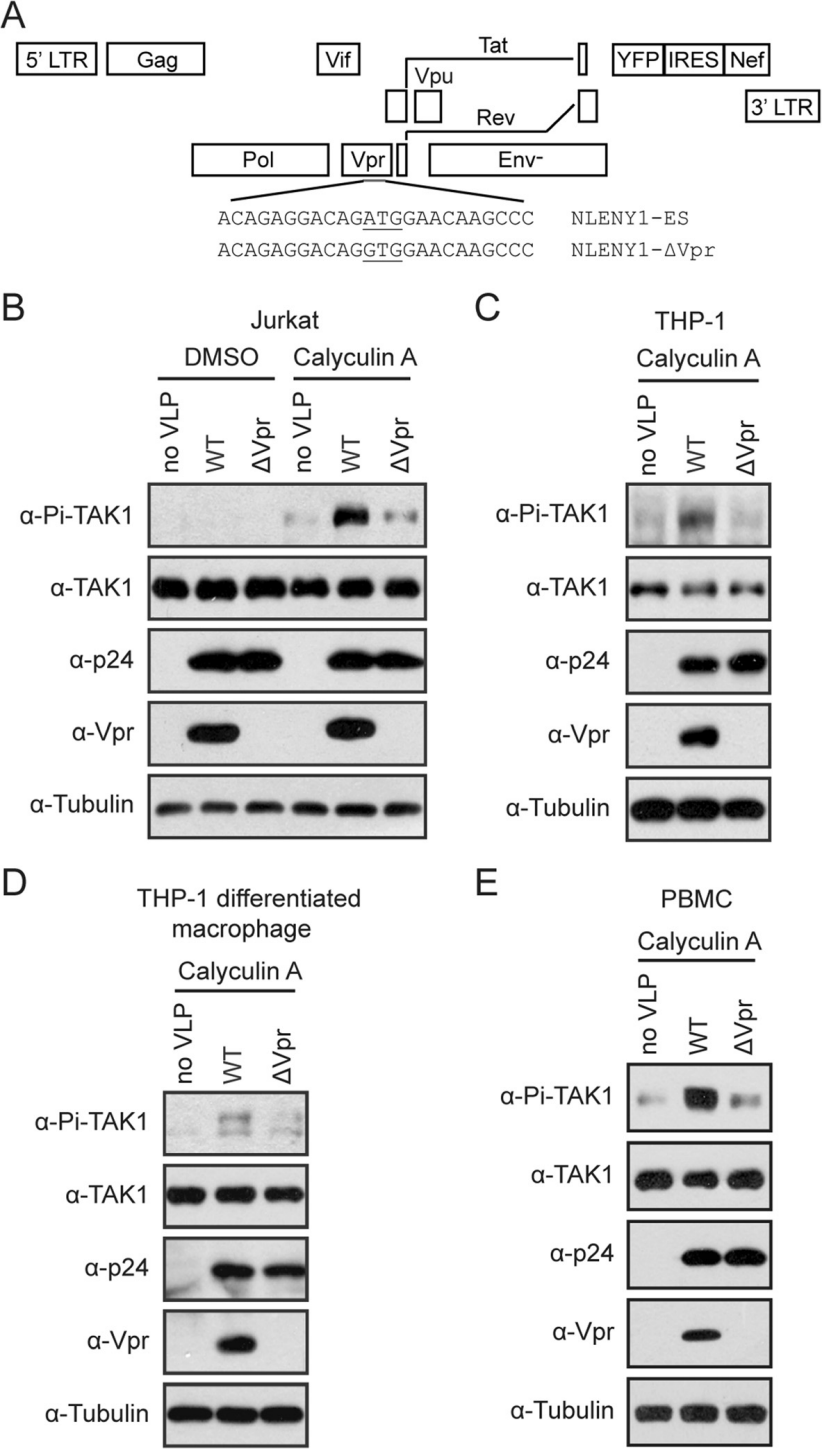
**Vpr enhances the phosphorylation of TAK1 following HIV-1 infection. (A)** Schematic representation of NLENY1-ES-IRES and NLENY1-ΔVpr viruses. A mutated start codon of vpr gene was introduced in NLENY1-ES to generate NLENY1-ΔVpr. **(B-D)** Virion-associated Vpr enhances the phosphorylation of TAK1 on Thr-187 in HIV-1 permissive cells. A total of 2 × 10^6^ Jurkat cells **(B)**, THP-1 cells **(C)**, and THP-1 differentiated macrophage-like cells **(D)** were infected with VSV-G pseudotyped WT or ΔVpr viruses equivalent to 500 ng p24 in the presence of 5 μg/ml polybrene by spinoculation at 300 xg for 30 min. After another 1.5 hours, cells were pretreated with 15 nM Calyculin A for 5 min. Then cells were harvested by centrifugation at 1000 × g for 3 min at 4°C, washed twice with ice-cold 1xphosphate-buffered saline, lysed in 70 μl lysis buffer, and chilled on ice for 30 min with frequent agitation. Whole cell lysates were subjected to Western blotting and probed with anti-phospho-TAK1 (Thr-187), rabbit anti-TAK1, anti-p24, anti-Vpr, and anti-Tubulin antibodies. **(E)** Human peripheral blood mononuclear cells (PBMCs) were isolated from healthy blood donors. PBMCs were activated with phytohemagglutinin (PHA; 5 mg/ml) and IL-2 (20 U/ml) for 24 h, followed by infection with VSV-G pseudotyped WT or ΔVpr (equivalent to 500 ng p24) in the presence of 5 μg/ml polybrene by spinoculation at 300 xg for 30 min. After two hours, cells were pretreated with 15 nM Calyculin A for 5 min. Whole cell lysates were examined in Western blotting with the indicated antibodies.

Since TAK1 is the upstream kinase for both NF-κB and AP-1 signaling pathways, we first asked whether HIV-1 virion-associated Vpr activates NF-κB and AP-1 by modulating the activity of TAK1. To answer this question, we monitored TAK1 activation by measuring its auto-phosphorylation at the major phosphorylation site Thr-187 [31]. We utilized the same amounts of VSV-G pseudotyped NLENY1-ES-IRES (WT for short) and NLENY1-ΔVpr (ΔVpr for short) viruses (Figure 1A) to infect the CD4+ T cells called Jurkat and harvested the infected cells 2 hours after infection before viral proteins were newly synthesized. Cells were treated with 15 nM Calyculin A (a serine/threonine phosphatase inhibitor of both PP1 and PP2A families) for 5 min prior to harvest in order to preserve the phosphorylated TAK1 molecules. Levels of total endogenous TAK1 and its phosphorylated form were assessed by Western blotting. Results of Figure 1B confirmed that the WT, not ΔVpr viruses, carried Vpr protein. In contrast to the ΔVpr virus, infection of the WT virus significantly increased phosphorylation of endogenous TAK1 (Figure 1B).

**Figure 2:**
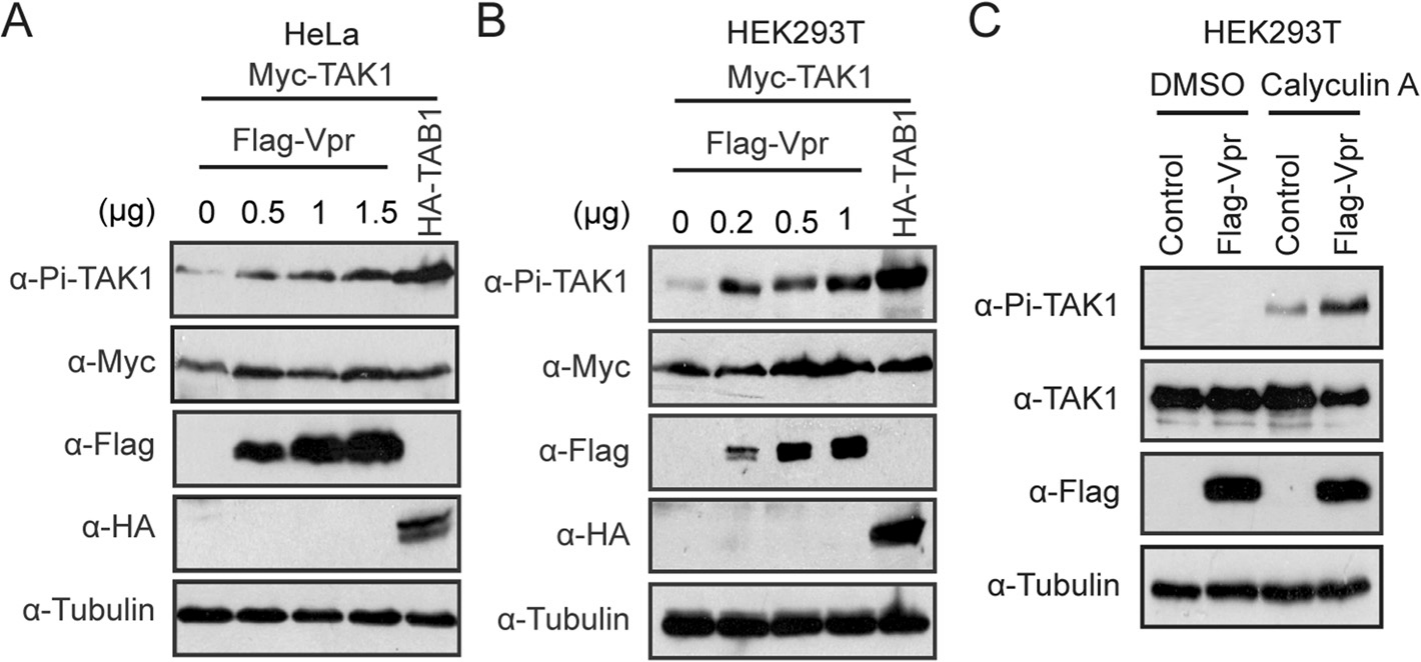
**Vpr enhances the phosphorylation of TAK1 in HeLa and 293T cells. (A-B)** Vpr induces the phosphorylation of exogenous TAK1 in a dose-dependent manner. HeLa cells (0.25 × 10^6^) **(A)** and HEK293T (0.5 × 10^6^) **(B)** were transfected with 0.3 μg Myc-TAK1 and increasing amounts of Flag-Vpr, or 0.3 μg HA-TAB1. Different amounts of Vpr plasmid were transfected into HEK293T cells (0, 0.2, 0.5 and 1 μg) and HeLa cells (0, 0.5, 1, and 1.5 μg). Forty-eight hours after transfection, whole cell lysates were harvested and probed with indicated antibodies. **(C)** Vpr induces the phosphorylation of endogenous TAK1. HEK293T cells were transfected with 1 μg Flag-Vpr or an empty vector. After forty-eight hours, cells were pretreated with DMSO or 20 nM Calyculin A for 5 min. Whole cell lysates were subjected to Western blotting and probed with indicated antibodies.

To rule out the possible effect of viral genome, we tested Vpr-induced phosphorylation of TAK1 in the presence of a reverse transcriptase inhibitor named AZT (10 μM). The results showed that under AZT treatment, the WT virus, but not the ΔVpr virus, still greatly enhanced TAK1 phosphorylation (Additional file 1: Figure S1A). In addition, we also performed TAK1 phosphorylation assay with HIV-1 VLP. We first produced VSV-G pseudotyped HIV-1 VLP (without the genome) by transfecting HEK293T cells with the pVSV-G (encoding the glycoprotein of VSV), pLP1 (encoding Gag and Gag-Pol), pLP2 (encoding Rev) together with pcDNA3.1 or pcDNA3-Vpr DNA. Results of Western blotting showed that HIV-1 VLP (Vpr^+^), but not HIV-1 VLP (Vpr-) carried Vpr (Additional file 1: Figure S1B). Next, we used the same p24 amounts of HIV-1 VLP (Vpr+) or HIV-1 VLP (Vpr-) to infect Jurkat cells. Results of Additional file 1: Figure S1C show that phosphorylation of TAK1 was markedly increased following infection by the HIV-1 VLP (Vpr+) but not by HIV-1 VLP (Vpr-).Furthermore, we also observed an enhancement of TAK1 phosphorylation by Vpr in the monocyte cell line called THP-1 (Figure 1C) and THP-1 differentiated macrophage (Figure 1D). To validate this observation, we isolated peripheral blood mononuclear cells (PBMCs) from healthy donors and examined TAK1 phosphorylation upon infection by the wild type or ΔVpr HIV-1. The results showed that the WT virus, but not the ΔVpr virus, profoundly increased TAK1 phosphorylation (Figure 1E).In order to further demonstrate that it is Vpr, not other HIV-1 proteins, that caused TAK1 phosphorylation, we transfected HEK293T or HeLa cells with plasmid DNA that expressed Myc-TAK1 and Flag-Vpr. TAB1 overexpression is known to stimulate TAK1 phosphorylation and was used as a positive control. The results showed that Flag-Vpr markedly enhanced the phosphorylation of TAK1 in both HeLa and HEK293T cells (Figure 2A and B). We also observed Vpr-induced phosphorylation of endogenous TAK1 in HEK293T cells (Figure 2C). Taken together, these data suggest that Vpr increases the phosphorylation of TAK1 at the early stage of HIV-1 infection.

### Vpr associates with TAK1

**Figure 3:**
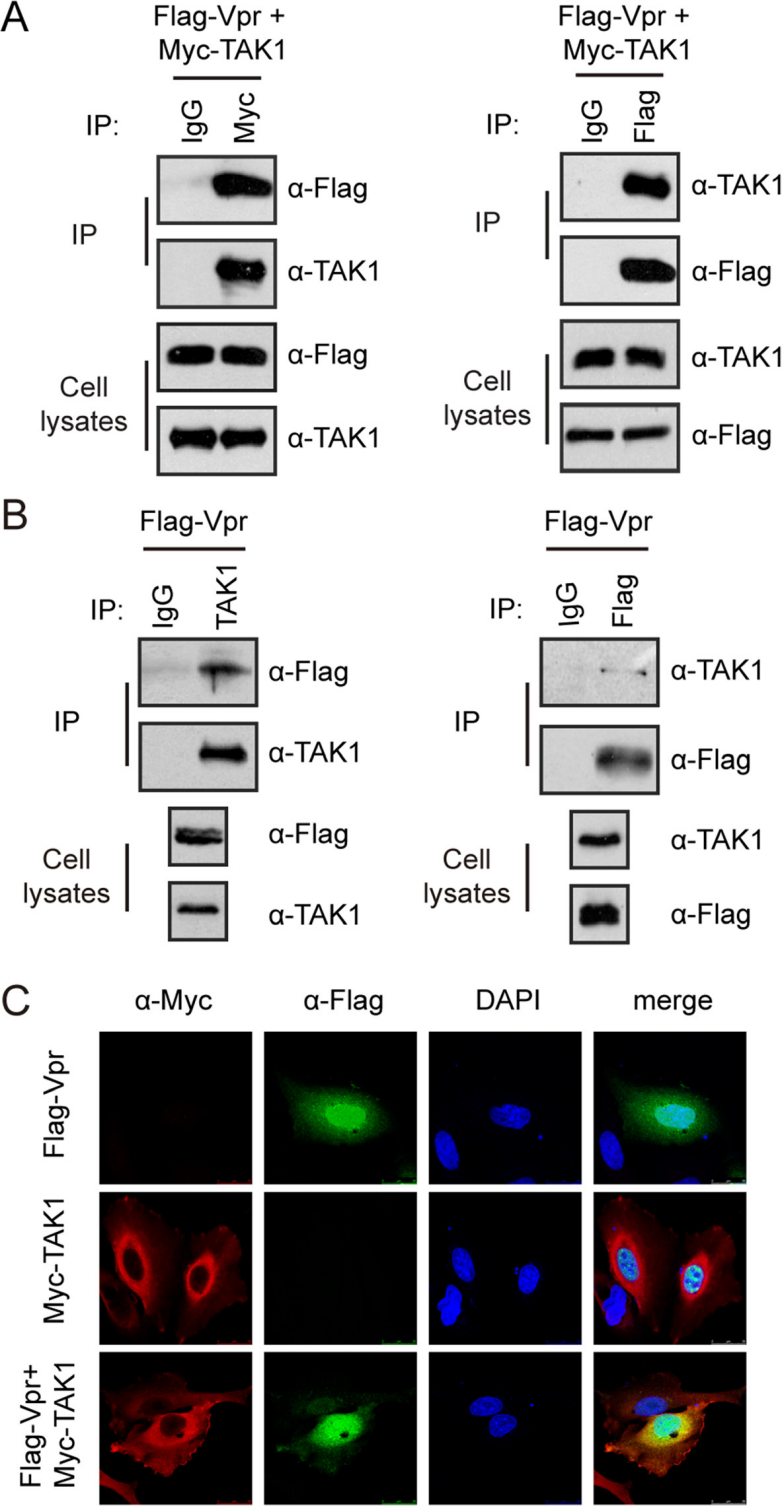
**Vpr associates with TAK1. (A)** HEK293T cells (4 × 10^6^) were co-transfected with Myc-TAK1 and Flag-Vpr. Co-immunoprecipitation was performed with indicated antibodies. Samples from both cell lysates and immunoprecipitates were subjected to Western blotting and probed with rabbit anti-TAK1 and mouse anti-Flag antibodies. **(B)** Vpr associates with endogenous TAK1. Lysates of Flag-Vpr-expressing HEK293T cells (4 × 10^6^) were immunoprecipitated with control mouse IgG and mouse anti-TAK1 (left) or anti-Flag (right) antibodies. Samples from both cell lysates and immunoprecipitates were subjected to Western blotting. **(C)** Vpr co-localizes at least in part with TAK1. HeLa cells (0.1 × 10^6^) were transfected with Flag-Vpr and/or Myc-TAK1 plasmid DNA. Indirect IFA was used to localize Vpr (with rabbit anti-Flag antibody and FITC-conjugated goat anti mouse secondary antibody) and TAK1 (with mouse anti-Myc antibody and TRITC-conjugated goat anti mouse secondary antibody). Nuclei were visualized with DAPI staining. Representative images are shown.

We next asked whether Vpr enhances TAK1 phosphorylation through association with TAK1. We first transfected HEK293T cells with plasmids expressing Flag-Vpr and Myc-TAK1, and performed co-immunoprecipitation experiments with anti-Myc or anti-Flag antibody. The results showed that Flag-Vpr was co-immunoprecipitated with Myc-TAK1 (Figure 3A). We also observed that Flag-Vpr was reciprocally immunoprecipitated with endogenous TAK1 (Figure 3B). In further support of the association between Vpr and TAK1, Vpr was found to partially co-localize with TAK1 in the perinuclear region (Figure 3C). Together, these data demonstrate an association of Vpr with TAK1.

### TRAF6 is required for Vpr-induced TAK1 phosphorylation

**Figure 4:**
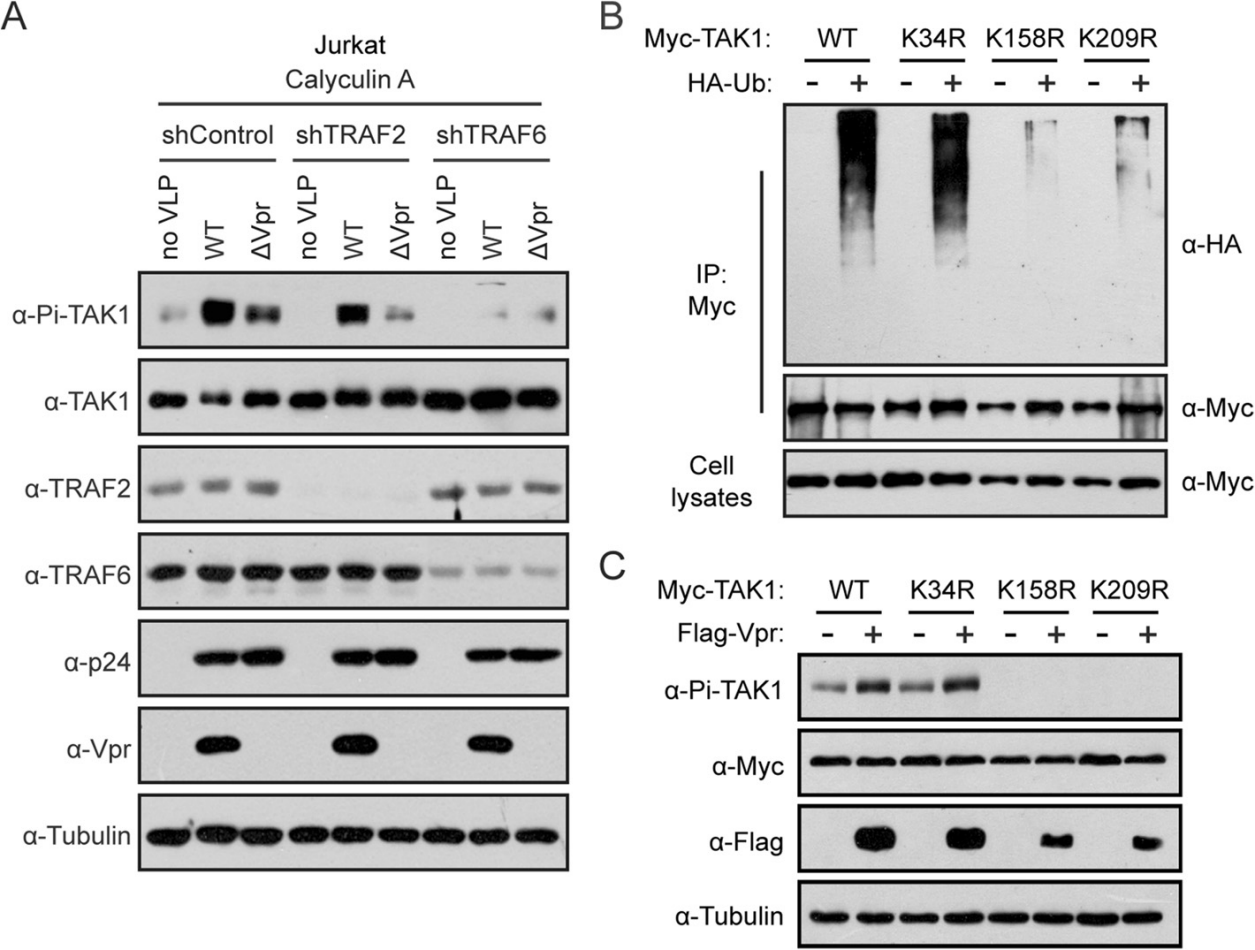
**TRAF6 is required for Vpr-induced TAK1 phosphorylation. (A)** Jurkat cells (2 × 10^6^) were transduced with retroviruses expressing shRNA targeting TRAF6, TRAF2 or a scrambled shRNA (shControl). After forty-eight hours, cells were exposed to VSV-G pseudotyped WT or ΔVpr (equivalent to 500 ng p24) in the presence of 5 μg/ml polybrene by spinoculation at 300 xg for 30 min. After two hours, cells were pretreated with 15 nM Calyculin A for 5 min. Whole cell lysates were examined in Western blotting with the indicated antibodies. **(B)** HEK293T cells (4 × 10^6^) were co-transfected with wild type TAK1 or its mutants (K34R, K158R, K209R) with or without HA-Ub DNA constructs. Forty-eight hours after transfection, cells were collected and denatured by boiling with 1% SDS, followed by immunoprecipitation with anti-Myc antibody. Polyubiquitination of TAK1 was detected with anti-HA antibody. Samples from both cell lysates and immunoprecipitates were probed with anti-TAK1 antibody. **(C)** Polyubiquitination of TAK1 is required for Vpr-induced TAK1 phosphorylation. HEK293T cells (0.5 × 10^6^) were transfected with 0.3 μg Myc-TAK1 or its mutants (K34R, K158R, K209R) along with empty vector or 0.5 μg Flag-Vpr. After forty-eight hours, whole cell lysates were subjected to western blotting and probed with indicated antibodies.

Given the dependence of TAK1 phosphorylation on TRAF6-mediated TAK1 polyubiquitination [41], we asked whether TRAF6 is needed for Vpr-induced TAK1 phosphorylation. To answer this question, we first generated two lentiviral vectors that express TRAF2- or TRAF6-specific shRNA. A non-specific scrambled shRNA was used as the control. We then examined whether virion-associated Vpr was still able to enhance TAK1 phosphorylation when TRAF6 or TRAF2 was knocked down in Jurkat cells. Results of Western blotting showed that endogenous TRAF6 and TRAF2 were effectively knocked down (Figure 4A). Knockdown of endogenous TRAF6 blocked the phosphorylation of TAK1 and ablated the stimulating effect of Vpr (Figure 4A). In contrast, knockdown of TRAF2 did not prevent Vpr from activating TAK1. These data indicate that TRAF6 is required for Vpr-induced TAK1 phosphorylation. In order to further demonstrate the dependence of Vpr-stimulated TAK1 phosphorylation on TAK1 polyubiquitination, we mutated each of the ubiquitin acceptor sites in TAK1, including Lys-34, Lys-158, and Lys-209 that have been reported in the literatures [41,43-47]. The results of Figure 4B showed that mutations K158R and K209R, but not K34R, abrogated TAK1 polyubiquitination. This defect in polyubiquitination correlated with the loss of TAK1 phosphorylation. Importantly, as opposed to increasing the phosphorylation of wild type and K34R TAK1, Vpr was unable to render the K158R and K209R mutants phosphorylated (Figure 4C). Together, we conclude that Vpr promotes TAK1 phosphorylation through modulating TRAF6-mediated polyubiquitination of TAK1.

### Vpr increases the polyubiquitination of TAK1

**Figure 5:**
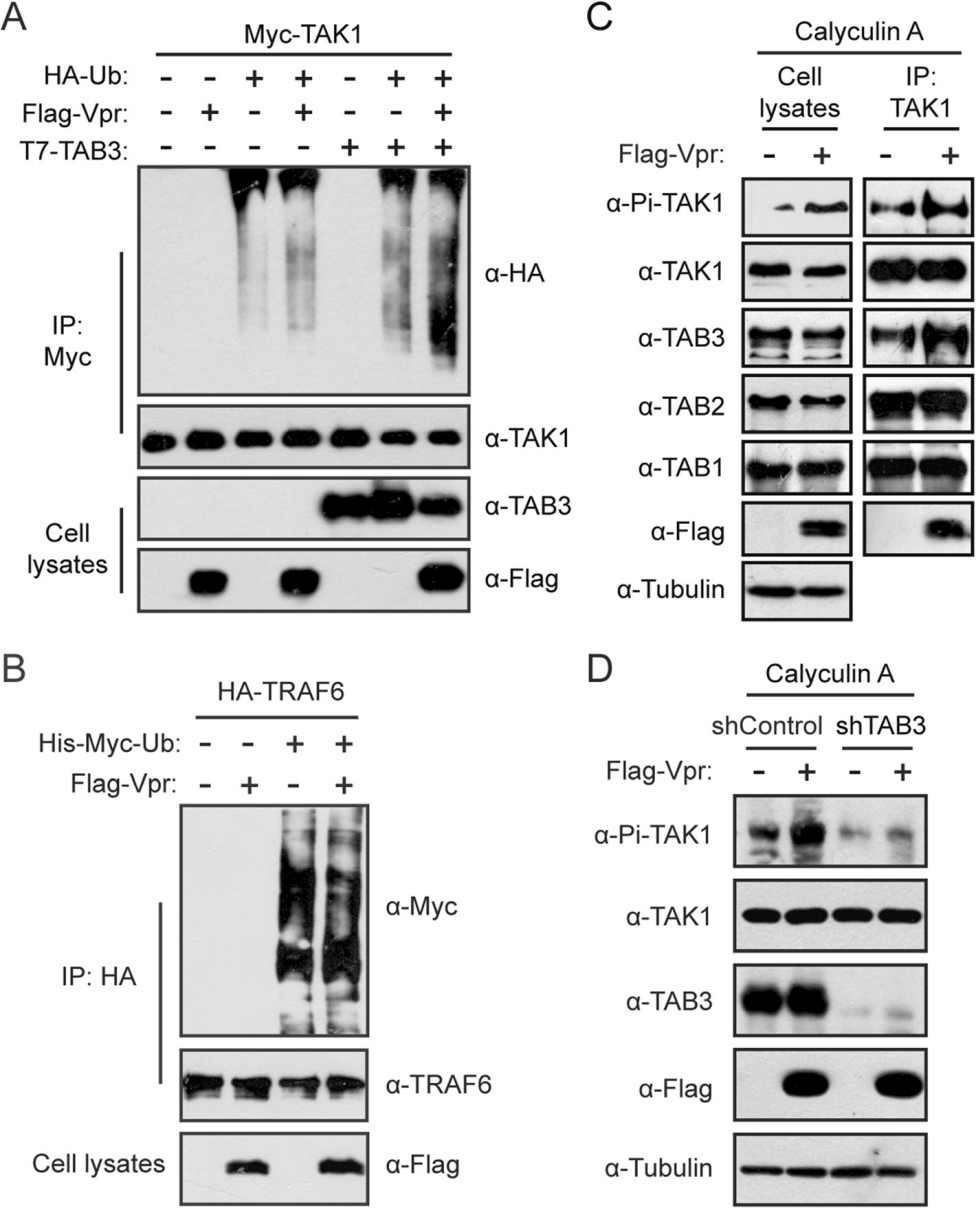
**Vpr increases the polyubiquitination of TAK1. (A)** Vpr synergizes with TAB3 to induce the polyubiquitination of TAK1. HEK293T cells (4 × 10^6^) were transfected with Myc-TAK1 together with Flag-Vpr, T7-TAB3 or HA-Ub. Forty-eight hours after transfection, cells were harvested and polyubiquitination of the immunoprecipitated TAK1 was detected in Western blotting with anti-HA antibody. **(B)** Vpr does not affect the auto-ubiquitination of TRAF6. HEK293T cells (4 × 10^6^) were transfected with HA-TRAF6 together with Vpr or Ub DNA. Cell lysates were immunoprecipitated with anti-HA antibody. Samples from both cell lysates and immunoprecipitates were probed with indicated antibodies. **(C)** Vpr promotes the association between TAK1 and TAB3. A total of 1 × 10^7^ HEK293T cells were transfected with an empty vector or Flag-Vpr DNA construct. After forty-eight hours, cells were pretreated with 20 nM Calyculin A for 5 min. Then whole cell lysates were immunoprecipitated with anti-TAK1 antibody. Samples from both cell lysates and immunoprecipitates were probed with rabbit anti-TAK1, anti-TAB1, anti-TAB2, anti-TAB3, anti-Flag, and anti-Tubulin antibodies. **(D)** TAB3 is involved in Vpr-induced phosphorylation of TAK1. The control and TAB3-knockdown HeLa cell lines (0.25 × 10^6^) were transfected with 1 μg empty vector or Flag-Vpr DNA constructs. After 48 hours, cells were pretreated with 20 nM Calyculin A for 5 min before cells were collected and analyzed by Western blotting with indicated antibodies.

Given the association of TAK1 polyubiquitination with Vpr-induced TAK1 phosphorylation, we postulate that Vpr may regulate TAK1 activity through modulating TAK1 polyubiquination. Indeed, expression of Vpr modestly increased the level of TAK1 polyubiquitination (Figure 5A), which is comparable to the increase that was caused by the overexpression of TAB3 that is responsible for recruiting TAK1 to the K63-linked polyubiquitin chains. Interestingly, co-expression of Vpr and TAB3 further increased TAK1 polyubiquitination (Figure 5A).This stimulating effect of Vpr on TAK1 polyubiquitination may result either from augmenting the enzymatic activity of TRAF6 or facilitating the recruitment of TAB2 or TAB3 to TAK1. It is known that TRAF6 undergoes auto-polyubiquitination. Results of Figure 5B showed that Vpr had no effect on this event, suggesting that the enzymatic activity of TRAF6 is not affected by Vpr. We next immunoprecipitated TAK1 and examined the presence of other components of the TAK1 complex. The results showed that Vpr markedly increased the level of TAB3 that was associated with TAK1, and had no effect on TAB1 and TAB2 (Figure 5C). This increase in TAB3 recruitment correlated with enhanced phosphorylation of TAK1 (Figure 5C). Since TAB3 interacts with both TAK1 and TRAF6, we conclude that Vpr augments the interaction of TAB3 with TAK1, consequently increases TRAF6-mediated polyubiquination and phosphorylation of TAK1. This key role of TAB3 in Vpr-induced phosphorylation of TAK1 is further demonstrated by the results of Figure 5D showing that knockdown of TAB3 in HeLa cells drastically reduced TAK1 phosphorylation and, more importantly, prevented Vpr from upregulating TAK1 phosphorylation.

### Activation of TAK1 by Vpr stimulates the NF-κB and AP-1 signaling

**Figure 6:**
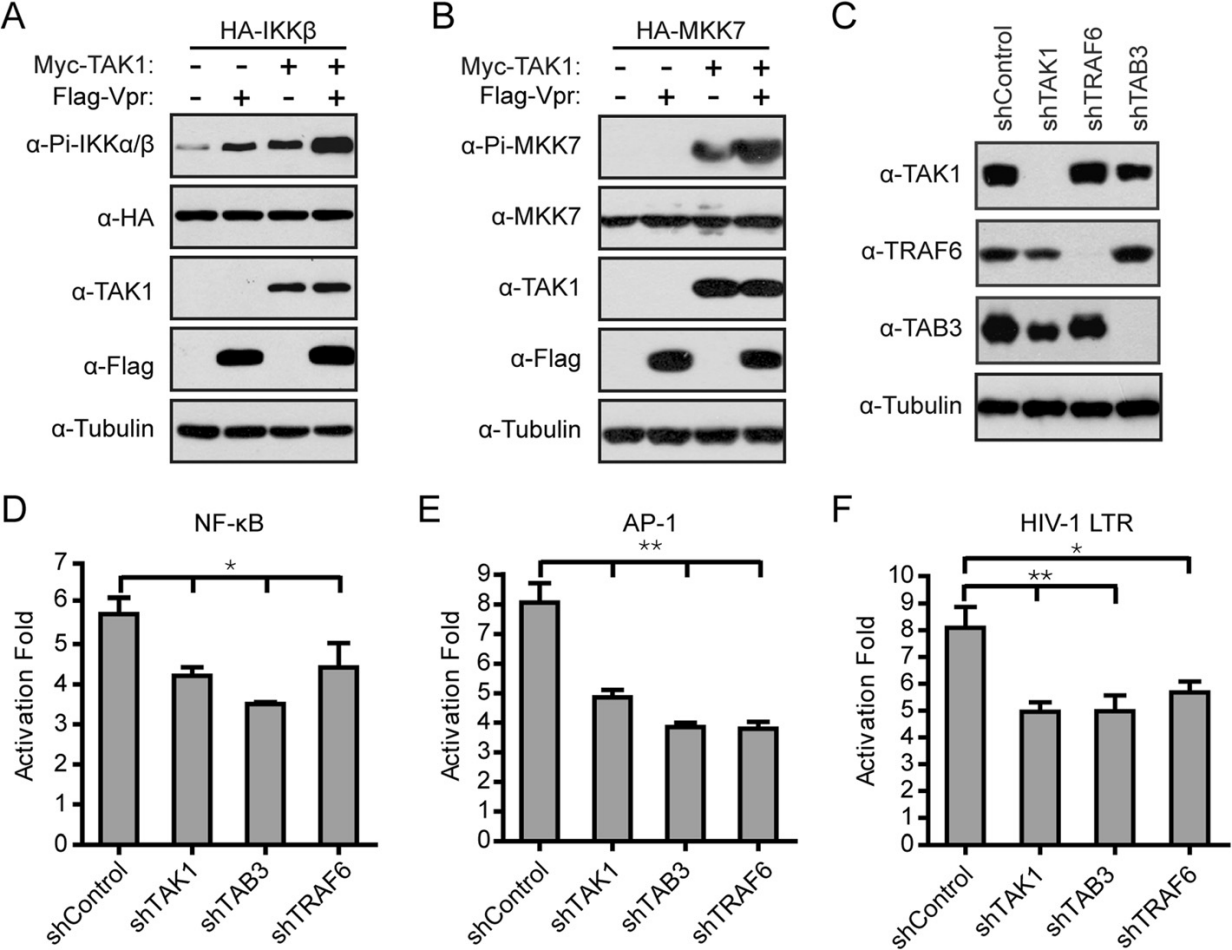
**TAK1 is required for Vpr-induced activation of NF-κB and AP-1 signaling. (A-B)** Vpr promotes TAK1-mediated phosphorylation of IKKβ and MKK7. HEK293T (0.5 × 10^6^) were transfected with 0.3 μg HA-IKKβ **(A)** or 0.5 μg HA-MKK7 **(B)**, with or without Myc-TAK1 and Flag-Vpr DNA constructs. After forty-eight hours, whole cell lysates were subjected to Western blotting and probed with indicated antibodies. **(C)** Expression of TAK1, TAB3, and TRAF6 in HeLa knockdown cell lines. Retroviruses coding shControl, TAK1, TAB3, or TRAF6 shRNA sequences, were stably transduced into HeLa cells. Cell lysates were immunoblotted with indicated antibodies. **(D-F)** Endogenous TAK1, TRAF6, and TAB3 are required for activation of NF-κB, AP-1, and HIV-1 LTR by Vpr. The aforementioned HeLa knockdown cell lines (0.1 × 10^6^) were transfected with vector or Flag-Vpr along with κB **(D)**, AP-1 **(E)**, or HIV-1 LTR **(F)** luciferase reporter plasmids. After forty-eight hours, luciferase activities were measured. The activation fold by Vpr was calculated by dividing the luciferase activity from Vpr-transfected cells by the luciferase activity from vector-transfected cells. The results shown are the averages of three independent experiments. The error bars indicate standard deviations. *P < 0.05, **P < 0.01 (paired *t* test).

IKKβ and MKK7 are two substrates of TAK1. Since Vpr activates TAK1, we expected that Vpr increases the phosphorylation of IKKβ and MKK7 by TAK1. Indeed, overexpression of either Vpr or TAK1 upregulated the phosphorylation of IKKβ (Figure 6A). A higher level of IKKβ phosphorylation was detected when Vpr and TAK1 were co-expressed (Figure 6A). Although we did not detect an increase in MKK7 phosphorylation when Vpr itself was overexpressed, significant phosphorylation of MKK7 was observed with TAK1 overexpression and this level of MKK7 phosphorylation was further increased by Vpr (Figure 6B). Together, these data indicate that activation of TAK1 by Vpr leads to activation of IKKβ and MKK7.It is known that phosphorylation of IKKβ and MKK7 causes relocation of NF-κB and AP-1 into the nucleus, respectively, and leads to upregulation of genes whose promoters contain the binding sequences for NF-κB or AP-1. It is thus not surprising that Vpr enhances the expression of luciferase that reports the activation of NF-κB or AP-1 (Figure 6D and E, column 1). Importantly, knockdown of TAK1, TRAF6, or TAB3 (Figure 6C) significantly diminished the ability of Vpr to activate NF-κB- or AP-1-dependent luciferase expression (Figure 6D and E). We also observed that activation of HIV-1 LTR promoter by Vpr was also attenuated as a result of knockdown of TAK1, TRAF6 or TAB3 (Figure 6F).

We also tested the role of TAK1 on the HIV-1 replication in TAK1-knockdown THP-1 cells. We first mutated the translation start codon of Vpr in the context of the HIV-Luc proviral DNA and generated HIV-Luc ΔVpr. We then produced VSV-G pseudotyped HIV-1 virus particles by transfecting HEK293T cells. As expected, the HIV-Luc virus, but not HIV-Luc ΔVpr, expressed Vpr (Additional file 1: Figure S2A). We used the same amount of these two viruses (equivalent to 2 ng p24) to infect TAK1-knockdown THP-1 cells (Additional file 1: Figure S2B). The infection of HIV-1 was assessed by measuring the luciferase activities of infected cells. Depleting endogenous TAK1 diminished the replication of HIV-Luc virus by 2-fold, whereas the infection of HIV-Luc ΔVpr virus was not affected (Additional file 1: Figure S2C). Together, these data demonstrate that TAK1 is involved in the replication of HIV-1 in a Vpr-dependent manner.

**Figure 7:**
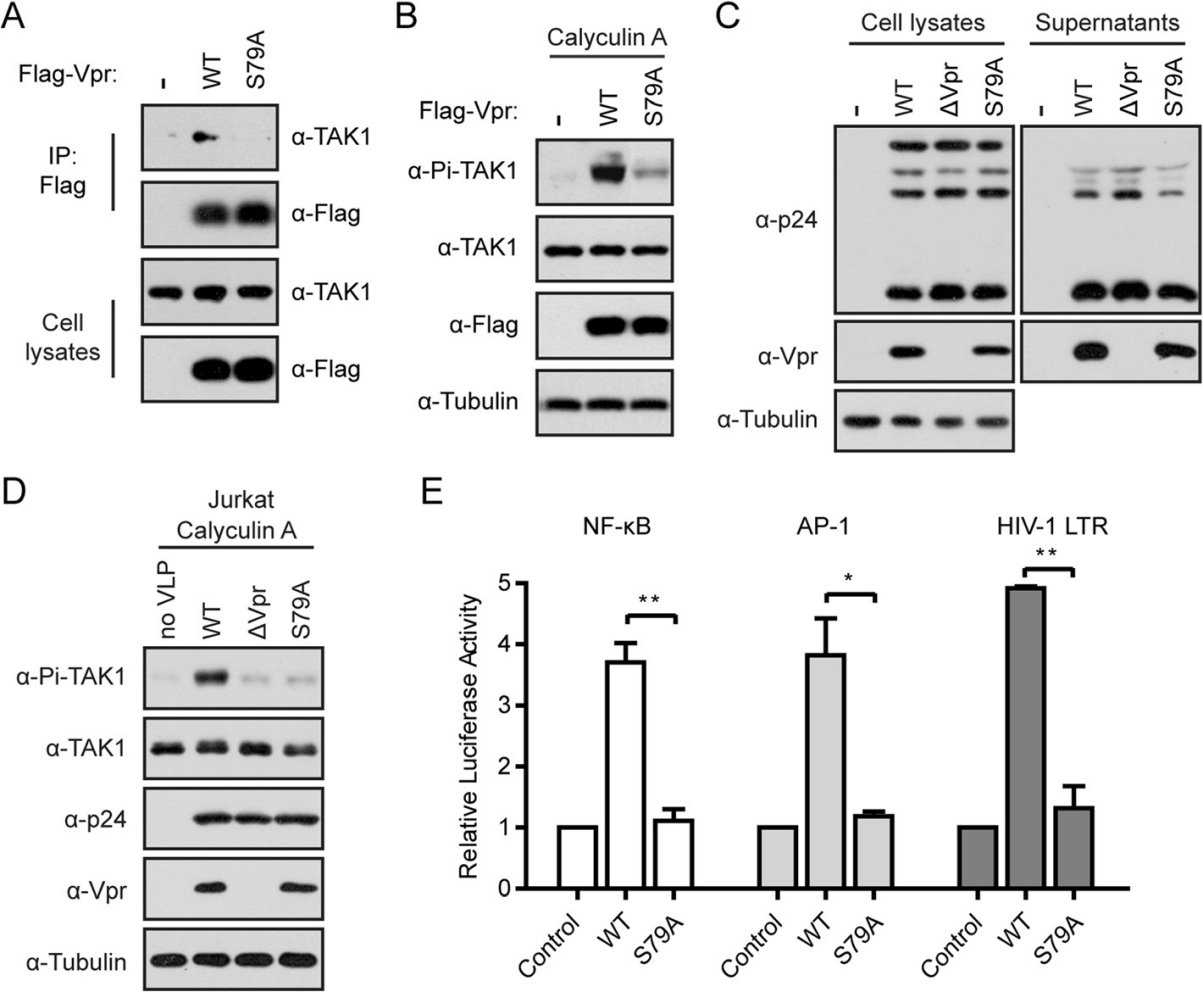
**The S79A mutant of Vpr is unable to activate NF-κB and AP-1. (A)** Vpr S79A failed to associate with endogenous TAK1. HEK293T cells (4 × 10^6^) were transfected with wild type Flag-Vpr or its mutant S79A. After forty-eight hours, cell lysates were immunoprecipitated with anti-Flag antibody. Samples from both cell lysates and immunoprecipitates were probed with rabbit anti-TAK1 and anti-Flag antibodies. **(B)** Vpr S79A was unable to enhance the phosphorylation of TAK1. A total of 0.5 × 10^6^ HEK293T cells were transfected with wild type Flag-Vpr or its mutant S79A. After forty-eight hours, cells were pretreated with 20 nM Calyculin A for 5 min. Whole cell lysates were subjected to Western blotting and probed with indicated antibodies. **(C)** HEK293T cells (4 × 10^6^) were transfected with 1 μg pVSV-G along with 8 μg NLENY1-ES (WT), NLENY1-ΔVpr (ΔVpr), or NLENY1-S79A (S79A) by PEI. The cell lysates and viral supernatants were subjected to Western blotting with the indicated antibodies. **(D)** Jurkat cells (2 × 10^6^) were infected with VSV-G pseudotyped WT, ΔVpr, or S79A equivalent to 500 ng p24 in the presence of 5 μg/ml polybrene by spinoculation at 300 xg for 30 min. After two hours, cells were pretreated with 15 nM Calyculin A for 5 min. Whole cell lysates were subjected to Western blotting with the indicated antibodies. **(E)** Ser-79 is required for Vpr-induced NF-κB, AP-1, and HIV-1 LTR activation. HeLa cells (0.1 × 10^6^) were transfected with Flag-Vpr or its S79A mutant along with κB, AP-1, or HIV-1 LTR luciferase reporter plasmid. After forty-eight hours, luciferase activities were measured. The results shown are the averages of three independent experiments. The error bars indicate standard deviations. *P < 0.05, **P < 0.01 (paired *t* test).

In further support of the dependence on TAK1 for Vpr to activate NF-κB and AP-1, we examined the effect of a Vpr mutant called S79A, known to be crucial for the function of Vpr [49]. We found that S79A lost its interaction with TAK1 (Figure 7A). Furthermore, S79A was unable to enhance TAK1 phosphorylation (Figure 7B). To further confirm this finding, we mutated sernine 79 of Vpr in the context of the pNLENY1-ES (WT) proviral DNA clone and generated pNLENY1-ES S79A. We then produced VSV-G pseudotyped HIV-1 virus particles by transfecting HEK293T cells with proviral DNA clones together with the pVSV-G DNA (Figure 7C). Next, we used the same amounts of viruses WT, ΔVpr and S79A to infect Jurkat cells and examined the phosphorylation of TAK1. The S79A virus was unable to induce the phosphorylation of TAK1 (Figure 7D). In addition, S79A failed to stimulate NF-κB- or AP-1-dependent luciferase expression (Figure 7E). Together, we conclude that activation of TAK1 by Vpr leads to stimulating NF-κB and AP-1-dependent gene expression.

## Discussion

HIV-1 Vpr plays an important role in viral replication and pathogenesis. It has been well established that Vpr can activate both NF-κB and AP-1 signaling [16-19,50], but how Vpr regulates these cascades remains unclear. In this study, we have identified TAK1 as an important player in Vpr-mediated NF-κB and AP-1 signaling. We present data showing that virion-associated Vpr enhances the phosphorylation of TAK1 by increasing TAK1 polyubiquitination through strengthening the association of TAB3 with TAK1.

Many viruses have been reported to modulate the activity of TAK1 and thereby the NF-κB and AP-1 pathways in order to benefit viral replication. For example, the X protein of hepatitis B virus, LMP1 of Epstein-Barr virus, vGPCR of KHSV, ICP0 of herpes simplex virus type 1, and Tax of human T cell leukemia virus type 1 activate NF-κB or AP-1 signaling by targeting TAK1 [51-55]. TAK1 has also been identified as an important upstream signaling molecule that RSV assimilates to activate NF-κB and AP-1 [56]. Recently, it was reported that HIV-1 gp41 activates NF-κB signaling via a direct interaction with TAK1, which leads to activation of CD4+ T lymphocyte cells and facilitate HIV-1 replication [48]. For the first time, we report that HIV-1 virion-associated Vpr associates with TAK1 and enhances the phosphorylation of TAK1 (Figure 1). We noted that knockdown of TAK1 does not completely prevent Vpr from activating NF-κB or AP-1 (Figure 6D). This is likely because Vpr may also activate these two important transcription factors by modulating other signaling pathways. An incoming HIV-1 particle carries up to 250-700 copies of Vpr protein and only about 7-16 envelope trimmers [6,57,58]. Compared with the small number of gp41, the relatively abundant virion-associated Vpr protein is expected to be more effective in activating TAK1 and NF-κB. Indeed, using VSV-G pseudotyped HIV-1 to infect cells, we found that Vpr alone, in the absence of gp41, is able to activate TAK1 (Figure 1B-E).

Recent studies showed that two cellular restriction factors, TRIM5 and tetherin, act as innate immune sensors to detect retroviral infection. Both restriction factors activate TAK1, followed by stimulating the expression of NF-κB and AP-1-dependent genes [59,60]. Given that HIV-1 benefits from activation of TAK1 by Vpr at the early stage of HIV-1 infection, it is possible that TAK1 is activated via different mechanisms by Vpr or TRIM5/tetherin. As a result, the downstream signaling and resulting gene expression profiles differ in regard to either facilitate HIV-1 replication (in the case of Vpr) or suppress HIV-1 replication (in the case of TRIM5/tetherin). It is also possible that Vpr competes with TRIM5/tetherin for binding to TAK1 and therefore counters innate immune response.

In addition to regulating the phosphorylation of TAK1, we also found that Vpr can enhance the polyubiquitination of TAK1 by recruiting TAB3 (Figure 5). It is possible that Vpr may facilitate the formation of TAK1-TAB2/3 complexes, and therefore enhances TRAF6-mediated K63-linked polyubiquitination of TAK1. We also found that polyubiquitination of TAK1 is crucial for its phosphorylation and activation (Figure 4B and C). Several lysine residues within TAK1, including Lys-34, Lys-158, and Lys-209, have been reported to be ubiquitinated in response to various stimuli [41,43-47]. Of these three lysines, Lys-158 and Lys-209 are located in the kinase domain of TAK1 and are essential in the polyubiquitination and activation of TAK1, with Lys-158 playing a major role (Figure 4B). These data support the notion that polyubiquitination of TAK1 may cause conformational change of the TAK1 that exposes the critical phosphorylation site (Thr-187) in the activation loop of TAK1 for auto-phosphorylation.

Several viral proteins have been reported to co-opt cellular signaling molecules. For example, HTLV-1 Tax protein constitutively activates NF-κB by directly interacting with NEMO in viral infected T lymphoma cell lines [61]. Recently, it was demonstrated that Tax interacts with TAK1 and recruits IKK complex to TAK1 [62,63]. In a similar fashion, we previously showed that Vpr activates both canonical and noncanonical NF-κB signaling by enhancing the phosphorylation of IKK complex [50]. Results in this study further show that TAK1 is the upstream kinase that is activated by Vpr and subsequently acts on IKK/NF-κB pathway. Activation of both NF-κB and AP-1 signaling suffice to enhance LTR-dependent viral gene expression and up-regulate the expression of several cytokines, such as IL-6, IL-8, and CCL5 [16-18]. This may facilitate the virus to establish a productive infection at the very early stage.

## Conclusions

In summary, results of this study demonstrate the role of TAK1 in Vpr-induced NF-κB and AP-1 activation. Vpr itself is able to enhance the phosphorylation and polyubiquitination of TAK1, and as a result, activates HIV-1 LTR promoter. These results support an important role of Vpr in assisting HIV-1 to establish productive infection by elevating early viral gene expression through modifying the cellular environment.

## Methods

### Plasmids

The DNA constructs Myc-TAK1, HA-TAB1, HA-TRAF6, HA-IKKβ, 3 × NF-κB-Luc, 7 × AP-1-Luc, and HIV-1 LTR-Luc were described previously [50,55,64-66]. The DNA constructs T7-TAB3 were gifts from Giichi Takaesu (Center for Integrated Medicine Research, School of Medicine, Keio University, Japan) [26]. The pCDNA-Flag Vpr (Flag-Vpr) construct was kindly provided by Kuan-Teh Jeang (Molecular Virology Section, Laboratory of Molecular Microbiology, National Institutes of Allergy and Infectious Diseases, USA) [67]. Plasmid pcDNA-FLAG Vpr was constructed by PCR amplification of pNL4-3 Vpr and cloning of PCR products into pCDNA3.1 vector.

The DNA construct pcDNA3-Vpr was generated by overlap PCR using pCDNA-Flag Vpr as a template. The coding sequence of human full length MKK7 was amplified from HEK293T cDNA, then cloned into the pCMV-HA expression vector (Clontech).

Myc-TAK1 K34R, K158R, K209R and Flag-Vpr S79A were generated using PCR-based mutagenesis. The PCR primers are listed in Additional file 1: Table S1.

The pNLENY1-ES-IRES DNA construct was kindly provided by David Levy [68]. This HIV-1 DNA was derived from the NL4-3 strain by inserting two-stop codons into the envelope gene. The enhanced yellow fluorescent protein (YFP) sequence was inserted between the *env* and *nef* ORFs. An internal ribosome entry sequence (IRES) was inserted upstream of *nef* to direct the expression of Nef. All accessory genes are left intact. The HIV-1 Luc was a gift from Johnny J He [69]. This proviral DNA construct was derived from the NL4-3 strain with *env* gene inactivated and the firefly luciferase (Luc) gene in place of HIV-1 *nef*. The pNLENY1-ΔVpr and pHIV-Luc ΔVpr were derived from pNLENY1-ES-IRES or pHIV-Luc by mutating the start codon ATG of *vpr* gene [50]. The pNLENY1-S79A was generated with PCR-based mutagenesis using primers listed in Additional file 1: Table S1.

All DNA constructs used in this study were verified by sequencing.

### Cell culture and transfection

HeLa and HEK293T cells were maintained in Dulbecco’s modified Eagle’s medium (high glucose) supplemented with 10% FBS (Gibco), 100 U/ml penicillin/streptomycin (Invitrogen). Jurkat, THP-1 and human peripheral blood mononuclear cells (PBMCs) were maintained in RPMI 1640 supplemented with 10% FBS, 100 U/ml penicillin/streptomycin, 2.4 mM L-glutamine. Cells were maintained in a humidified atmosphere containing 5% CO_2_ at 37°C. HeLa and HEK293T were transfected by PEI (Polysciences) or Lipofectamine 2000 (Invitrogen) in accordance with the manufacturer’s instructions.

PBMCs were isolated from buffy coat from healthy blood donors by histopaque and percoll gradient centrifugation, and were activated with phytohemagglutinin (PHA; 5 mg/ml) (Sigma) and IL-2 (20 U/ml) (PeproTech) for 24 h before virus infection.

To differentiate THP-1 monocytes into macrophage-like cells, THP-1 cells were seeded at a concentration of 1 × 10^6^ cells/ml in 1.5 ml fresh cell culture medium containing 200 ng/ml tetradecanoylphorbol acetate (PMA) (Sigma). After twenty-four hours, cell culture media were changed to remove PMA. Then cells were rested for an additional 48 hours before use.

### Antibodies and reagents

Anti-phospho-TAK1 (Thr-187), rabbit anti-TAK1, anti-TRAF2, anti-TRAF6, anti-TAB1, anti-TAB2, anti-phospho-IKKα/β (Ser176/180), anti-phospho-MKK7 (Ser-271/Thr-275), and anti-MKK7 antibodies were purchased from Cell Signaling Technology. Anti-TAB3 antibody was from Abcam. Anti-Myc antibody, normal mouse IgG and normal rabbit IgG were from Millipore. Anti-Flag antibody (M2) was obtained from Sigma. Anti-HA, anti-α-Tubulin, and horseradish peroxidase-conjugated secondary antibodies were from Santa Cruz Biotechnology. Rabbit anti-Vpr antibody was provided by National Institutes of Health (NIH) AIDS Research and Reference Reagent Program. Mouse anti-p24 and anti-TAK1 were generated by immunizing mice with the corresponding full length proteins purified form *E. coli* BL21 (DE3). 4′, 6-diamidino-2-phenylindole (DAPI) and PMA were purchased from Sigma. Fluorescein-conjugated anti-mouse and anti-rabbit secondary antibodies were purchased from Jackson ImmunoResearch Laboratories. Serine/Threonine phosphatase inhibitor Calyculin A was purchased from Cell Signaling Technology.

### Generation of stably transduced cell lines

First, shRNA oligos targeting TAK1, TAB3, and TRAF6 were designed by shRNA Sequence Designer (Clontech). Double-stranded oligonucleotides corresponding to the target sequences were cloned into the pSIREN-RetroQ(Clontech). The target sequences were as follows, shControl (without specific target in cells) (GACAGAACCAGAGGATAGA), TAK1 (AGGCAAAGCAACAGAGTGA), TAB3 (CCTTCACCCATCAGTAATC), TRAF6 (GCCTGGATTCTACACTGGCAAA), and TRAF2 (GGACCAAGACAAGATTGA A).

Then, retrovirus particles were prepared by transfecting HEK293T cells with 1 μg pMLV-Gag-Pol, 0.5 μg pVSV-G and 1 μg pSIREN-RetroQ DNA constructs. After forty-eight hours, supernatants were collected and centrifuged at 3,000 rpm to remove cell debris. HeLa cells were infected with the harvested virus particles in the presence of 5 μg/ml polybrene by spinoculation at 450 × g for 30 min at room temperature. Forty-eight hours after infection, cells were subcultured in selection medium containing 2 μg/ml puromycin (Sigma). Knockdown efficiency was assessed by Western blotting using specific antibodies.

### Viruses and infections

Infection with VSV-G pseudotyped HIV-1 viruses were performed as we previously described [50]. Briefly, the viruses were produced by transfecting HEK293T cells with plasmid DNA pVSV-G, and pNLENY1-ES-IRES or pNLENY1-ΔVpr. After forty-eight hours, the viral supernatants were collected, and the virus titer was determined by ELISA (Biomerieux) to quantify viral p24 amounts. The viruses (equivalent to 500 ng p24) were used to infect Jurkat, THP-1, THP-1 differentiated macrophage, and PBMC in the presence of 5 μg/ml polybrene by spinoculation in 1.6 ml fresh medium. After two hours, the infected cells were harvested to detect HIV-1-induced phosphorylation of endogenous TAK1.

### Protein phosphorylation analysis

At 2 hours post-infection or 48 hours post-transfection, cells were harvested by centrifugation at 1000 × g for 3 min at 4°C, washed twice with ice-cold 1xphosphate-buffered saline, and suspended in 70 μl lysis buffer (20 mM Tris (pH 7.4), 150 mM NaCl, 2 mM EDTA, 1% Triton-X-100, 1 mM sodium orthovanadate, 50 mM sodium fluoride, phosphatase inhibitor mixture tablet (Roche), protease inhibitor cocktail tablets complete, EDTA-free (Roche)), and chilled on ice for 30 min with frequent agitation. Cell lysates were collected by centrifugation at 13,000 × g for 10 min at 4°C. The supernatants were separated by SDS-PAGE and subjected to Western blotting.

### Immunoprecipitation

For protein-protein interaction analysis, HEK293T cells were washed twice with ice-cold 1xphosphate-buffered saline, and lysed in lysis buffer (50 mM Tris (pH 7.4), 150 mM NaCl, 2 mM EDTA, 3% Glycerol, 1% NP-40, protease inhibitor cocktail tablets complete, EDTA-free(Roche)). The cell lysates were sonicated and centrifuged at 13,000 × g for 10 min at 4°C. To detect the polyubiquitinated form of TAK1 and TRAF6, *in vivo* ubiquitination assay were performed as follows. Cells were lysed in 0.4 ml TNET buffer (20 mM Tris (pH 7.4), 150 mM NaCl, 5 mM EDTA, 1% Triton-X-100). Supernatants were denatured in 1% SDS by boiling for 15 min to remove noncovalently attached proteins, and then diluted to 0.1% SDS with regular TENT buffer. Then, supernatants were incubated with indicated antibody for 3 h at 4°C and rotated with Protein A-agarose (Millipore) for 3 h or overnight (for endogenous protein immunoprecipitation) at 4°C. After six times washes with lysis buffer, the immunoprecipitated materials were boiled in 40 μl 2 × SDS loading buffer and subjected to Western blotting.

### Western blotting

Cell lysates or immunoprecipitated materials were resolved by SDS-PAGE and transferred to a PVDF membrane (GE Healthcare). The membranes were blocked in 5% non-fat milk-TBS for 45 min at room temperature, and probed with indicated primary antibodies overnight at 4°C. After hybridizing the membranes with either goat anti-rabbit or goat anti-mouse secondary antibody, enhanced chemiluminescence reagents (Millipore) were used for signal detection with X-ray film.

### Immunofluorescence microscopy assay (IFA)

Indirect IFA was performed as we previously described [50]. HeLa cells grown on poly-lysine-coated glass slides were first fixed with 4% paraformaldehyde in PBS for 10 min and then permeabilized with 0.1% Triton X-100 in PBS for 10 min. After incubation in the blocking buffer containing 3% BSA and 6% skim milk, cells were stained with anti-Flag or anti-Myc primary antibodies (1:100 dilution, 2 h at room temperature) followed by FITC- or TRITC-conjugated secondary antibodies (1:100 dilution, 45 min at room temperature). DAPI was utilized to stain nuclei. Images were captured using Leica TCS SP5 laser scanning confocal microscope.

### Luciferase assay

HeLa RNAi cell lines were seeded at a concentration of 0.1 × 10^6^ cells/well. The next day, cells were transfected with indicated reporter gene and Flag-Vpr DNA along with the Renilla luciferase DNA. After forty-eight hours, luciferase activity was measured with a Dual-Luciferase reporter assay system according to the manufacturer’s instructions (Promega). The relative luciferase activity was calculated by dividing the firefly luciferase activity by the Renilla luciferase activity. Three independent transfection experiments were performed.

### Statistical analysis

Data were presented as mean values ± SD of at least three independent experiments. Statistical comparison of Vpr-transfected cells with matched control cells was performed using F-test and Student’s T-test. P values of <0.05 were considered to be statistically significant.

## Competing interests

The authors declare that they have no competing interests.

## Authors’ contributions

RL carried out experiments and analysed data. YL and RJ carried out experiments. JT, YG, and WQ conceived experiments and analysed data. RL and CL wrote the paper. All authors had final approval of the submitted and published versions.

## Supplementary Material

Additional file 1: Figure S1 Vpr increased the phosphorylation of TAK1 in the absence of viral genome. **Figure S2.** TAK1 is involved in the replication of HIV-1. **Table S1.** List of primers used in mutagenesis and cloning. Click here for file
