# Supplementary material for: HIV-1 Vpr stimulates NF-κB and AP-1 signaling by activating TAK1
Source: Retrovirology. 2014 Jun 9;11:45. doi: 10.1186/1742-4690-11-45 (PMC4057933; doi:10.1186/1742-4690-11-45)
Supplement: Additional file 1: Figure S1 — Vpr increased the phosphorylation of TAK1 in the absence of viral genome. Figure S2. TAK1 is involved in the replication of HIV-1. Table S1. List of primers used in mutagenesis and cloning. [file 1742-4690-11-45-S1.doc]

**Additional file**

**HIV-1 Vpr stimulates NF-κB and AP-1 signaling by activating TAK1**

Ruikang Liu ^a, 1^, Yongquan Lin ^a, 1^, Rui Jia^a^, Yunqi Geng^a^, Chen Liang^b,c,d^,

Juan Tan ^a^*, and Wentao Qiao^a^*


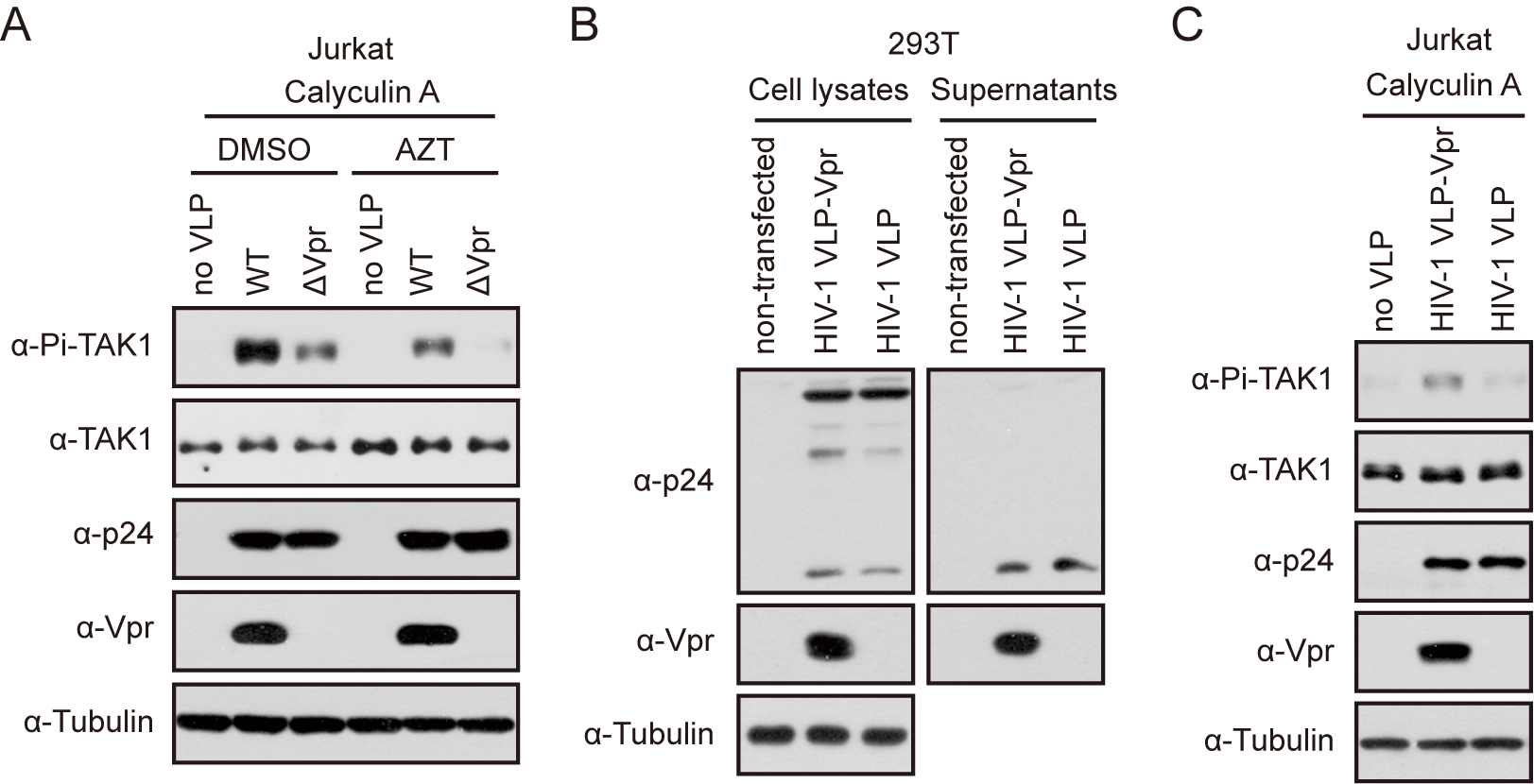


Fig. S1 Vpr increased the phosphorylation of TAK1 in the absence of viral genome

(A) A total of 2×10^6^ Jurkat cells were exposed to VSV-G pseudotyped WT or ΔVpr (equivalent to 500 ng p24) along with DMSO or 10 μM AZT (Sigma). Cells were spun at 300 xg for 30 min and incubated for another 1.5 hour before being harvested. After cells were pretreated with 15 nM Calyculin A for 5 min, whole cell lysates were prepared and examined in Western blotting with the indicated antibodies. (B) HEK293T cells were transfected with 1 µg pVSV-G, 5.5 µg pLP1 (invitrogen), 1.5 µg pLP2 (invitrogen), along with 4 µg pcDNA3.1 or 4 µg pcDNA3-Vpr (no Flag Tag) by PEI. Forty-eight hours after transfection, supernatants were collected and centrifuged at 3,000 rpm to remove cell debris. Cell lysates and viral supernatants were examined by Western blotting with the indicated antibodies. (C) Jurkat cells (2×10^6^) were infected with VSV-G pseudotyped HIV-1 VLP (Vpr+), or HIV-1 VLP (Vpr-) equivalent to 500 ng p24 in the presence of 5 μg/ml polybrene by spinoculation at 300 xg for 30 min. After two hours, cells were pretreated with 15 nM Calyculin A for 5 min. Whole cell lysates were subjected to Western blotting with the indicated antibodies.


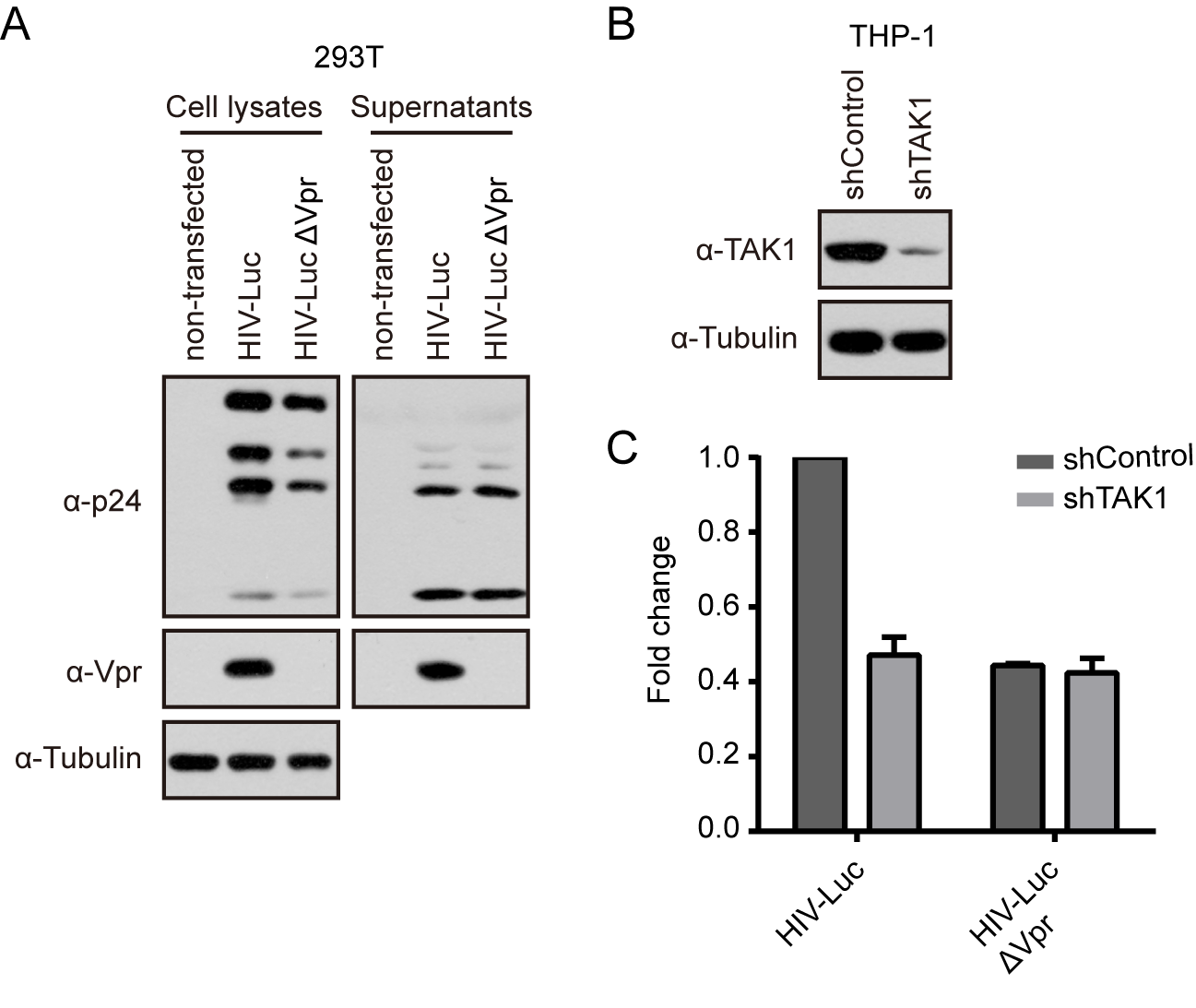


Fig. S2 TAK1 is involved in the replication of HIV-1

(A) HEK293T cells were transfected with 1 µg pVSV-G along with 8 µg HIV-Luc or HIV-Luc ΔVpr by PEI. The cell lysates and viral supernatants were examined in Western blotting with the indicated antibodies. (B) Retroviruses coding shControl, or TAK1 shRNA sequences, were stably transduced into THP-1 cells. Cell lysates were immunoblotted with rabbit anti-TAK1 and anti-tubulin antibodies. (C) The control and TAK1-knockdown THP-1 cell lines (0.4×10^6^) were infected with VSV-G pseudotyped HIV-Luc or HIV-Luc ΔVpr (equivalent to 2 ng p24). Forty-eight hours after infection, luciferase activities were measured. The fold change was calculated by dividing the values from HIV-Luc or HIV-Luc ΔVpr infected cells by the values from mock-infected cells. The results shown are the averages of three independent infection experiments. The error bars indicate standard deviations.

Table S1. List of primers used in mutagenesis and cloning.

|  | Forward primer | Reverse primer |
| --- | --- | --- |
| HA-MKK7 | TCTAGATCTCTATGGCGGCGTCCTCC | ATAGGTACCCTACCTGAAGAAGGGC |
| Myc-TAK1 K34R | GAAGAGATCGACTACAgGGAGATCGAGGTGGAAGAGGTTG | CAACCTCTTCCACCTCGATCTCCCTGTAGTCGATCTCTTC |
| Myc-TAK1 K158R | CGCTGATTCACAGGGACCTCAgGCCTCCAAACTTGCTGC | GCAGCAAGTTTGGAGGCCTGAGGTCCCTGTGAATCAGCG |
| Myc-TAK1 K209R | GGTAGCAATTACAGTGAAAgGTGTGATGTCTTCAGCTGGGG | CCCCAGCTGAAGACATCACACCTTTCACTGTAATTGCTACC |
| pCDNA3-Vpr | CTTGGTACCGAGCTCGGATCCACTAGTCCAGTGTGGTGGAATTCTGCAGATG | GCAACTAGAAGGCACAGTCGAGG |
| Vpr S79A | GAATTGGGTGTCGACATgcCAGAATAGGCGTTACTC | GAGTAACGCCTATTCTGGCATGTCGACACCCAATTC |
| ΔVpr | GTTAGGAAACTGACAGAGGACAGGTGGAACAAGCCCCAGAAGACCAAG | CTTGGTCTTCTGGGGCTTGTTCCACCTGTCCTCTGTCAGTTTCCTAAC |
